# IL-6 is a prognostic biomarker in patients with advanced esophageal squamous cell carcinoma received with PD-1 inhibitors

**DOI:** 10.3389/fimmu.2025.1569042

**Published:** 2025-05-13

**Authors:** Ping Huang, Manyi Zhao, Jianhong Xia, Hongliang Li, Junxia Sun, Xin Li, Chunsheng Yang, Guangyi Gao, Wenhang Zhou, Meifeng Zhong, Hongmei Yong

**Affiliations:** ^1^ Department of Medical Oncology, Huai’an Hospital Affiliated to Xuzhou Medical University, Huai’an, Jiangsu, China; ^2^ Department of Radiation Oncology, Huai’an Hospital Affiliated to Xuzhou Medical University, Huai’an, Jiangsu, China; ^3^ Department of Dermatology, Huai’an Hospital Affiliated to Xuzhou Medical University, Huai’an, Jiangsu, China; ^4^ Department of Pediatrics, Huai’an Hospital Affiliated to Xuzhou Medical University, Huai’an, Jiangsu, China

**Keywords:** esophageal squamous cell carcinoma, IL-6, PD-1 inhibitor, tumor microenvironment, prognosis

## Abstract

**Background:**

Due to the low efficacy rates, effective biomarkers are desperately needed to determine populations of advanced esophageal squamous cell carcinoma (ESCC) that may benefit from immune checkpoint inhibitor (ICI) treatment.

**Objectives:**

To explore the relationship between IL-6 and the esophageal cancer tumor immune microenvironment using online databases and esophageal cancer tissue microarrays and to investigate the predictive role of IL-6 for immunotherapy in esophageal squamous carcinoma patients through clinical study data.

**Methods:**

RNA-seq datasets of ESCC patients were obtained from TCGA, and the relationship between IL-6 and immune cells was discovered using TIMER 2.0 databases. CD8, IL-6, and PD-L1 expression in ESCC tissue microarrays were measured using immunohistochemistry, and then the tumor microenvironment was classified. Furthermore, blood specimens were collected from advanced ESCC patients before they received PD-1 inhibitors, and follow-up was conducted to gather clinical survival data. Based on IL-6 levels. We divided the population into the high and low IL-6 groups, comparing the efficacy and survival of the two groups.

**Results:**

IL-6 positively correlated with mRNA levels of PD-L1, negatively correlated with immune cells, and positively correlated with immunosuppressive cells. High IL-6 expression in tissues might make PD-1/L1 blockade therapy less effective. Individuals with higher baseline plasma IL-6 levels had significantly lower objective remission rates and inferior PFS and OS. Elevated baseline IL-6 was demonstrated to be an independent risk factor for the prognosis of advanced ESCC patients using PD-1 inhibitors, according to COX regression analysis.

**Conclusion:**

IL-6 overexpression correlates with the immunosuppressive tumor microenvironment in ESCC, and it can be a predictive biomarker in ESCC patients received with PD-1 inhibitors.

## Introduction

As per the 2020 global cancer statistics, esophageal cancer is the seventh most prevalent form of cancer worldwide (1). The most common pathological subgroup among Asians is esophageal squamous cell carcinoma (ESCC), which constitutes approximately 90% of new cases annually (2). In China, the incidence and mortality of ESCC rank sixth and fourth among all malignant tumors, respectively, which poses a huge economic and public health burden. Although early diagnosis and advancements in surgery, radiotherapy, chemotherapy, and targeted therapy have greatly improved survival rates for ESCC patients, effective treatment options remain limited for those with poor chemotherapy sensitivity or who cannot tolerate chemotherapy toxicity in advanced or metastatic stages (3).

As research in anti-tumor therapy advances, immunotherapy has become a mainstream treatment option. The discovery of immune checkpoint inhibitors (ICIs) has brought about a new wave of promising anti-cancer therapies. Most of these are PD-1 inhibitors, which obstruct the interaction between PD-1 receptors and their ligands, restoring T cells’ ability to identify and eradicate tumor cells (4). This approach has demonstrated promising therapeutic effects in treating solid tumors.

Patients with recurrent or metastatic ESCC are now treated with PD-1 inhibitors as the first line of therapy in China. Despite this, the efficacy rate for esophageal cancer remains suboptimal. Effective biomarkers are desperately needed to increase patient selection and ICI treatment accuracy, thereby improving the efficacy of immunotherapy for ESCC. The expression of programmed death receptor ligand 1 (PD-L1) in tumor tissues is a widely used criterion for choosing immunotherapy regimens, yet its predictive value for esophageal cancer outcomes is still debatable. Not all patients positive for PD-L1 benefit from immunotherapy, as effective responses can also occur in some PD-L1 negative patients (5). Furthermore, biomarkers, including microsatellite instability (MSI) and tumor mutation burden (TMB), have limited application due to their low positivity rates and high costs. Therefore, it is imperative to identify additional practical and reliable prognostic biomarkers.

Elevated cytokine levels are often detected in patients with inflammation and tumors. IL-6 is a common cytokine and a major mediator of inflammation, predominantly released by stromal cells, immune cells, and tumor cells in the tumor microenvironment. Through its downstream signaling pathway, tumor cells are encouraged to proliferate, survive, and invade (6). Numerous investigations have underscored the connection between increased IL-6 expression and a worse prognosis, as well as chemotherapy resistance, in patients with pancreatic cancer, non-small cell lung cancer (NSCLC), ovarian cancer, and other malignancies (7–10). Researchers believe that this may be due to the close relationship between IL-6 and tumor immunity. Research on IL-6 and tumor immunity has also significantly progressed in recent years. IL-6 can down-regulate the expression of MHC class II on dendritic cells through the STAT3 signaling pathway, inhibit their maturation, and weaken Th1 immunity (11). Xiao-Long Fu et al. (12) proved that IL-6 can induce the differentiation of normal macrophages into M2 type by activating STAT3 phosphorylation. Kun Fan et al. (13) confirmed that tumor-derived IL-6 in pancreatic cancer can promote FoxP3 expression and Treg differentiation. Therefore, IL-6 in the tumor microenvironment may affect the efficacy of tumor immunotherapy by affecting anti-tumor immunity.

Studies have shown that patients with high baseline plasma IL-6 levels have a worse response in patients with advanced non-small cell lung cancer (14) and liver cancer (15) treated with PD-1/PD-L1 inhibitors. When Alissa Keegan et al. studied cytokine changes in NSCLC patients treated with PD-1 inhibitors, they found that decreased IL-6 levels were associated with better PFS (16). However, the specific role that IL-6 plays in patients with advanced ESCC undergoing PD-1 inhibitor therapy is not yet known.

## Materials and methods

### Study design and patients

A total of 141 patients were included as study subjects. Our inclusion criteria were patients with advanced esophageal cancer who received at least 2 cycles of PD-1 inhibitors at the Huai’an Second People’s Hospital from August 2020 to August 2023. The patients listed below were excluded: I. Concurrent presence of other tumors; II;. Co-occurrence with inflammation, severe infection, or abnormal liver and kidney function; III. Coexistence with other conditions influencing IL-6 levels, such as autoimmune diseases, myocardial injury, heart failure, type 2 diabetes, or burn trauma; IV. Use of medications affecting IL-6 levels, such as statins; V. Lack of pre-treatment blood specimens and pre- and post-treatment imaging data. Baseline blood samples and clinical data, encompassing the age and gender of inclusion, Eastern Cooperative Oncology Group (ECOG) score, smoking history, tumor stage and site, number of metastatic organs, previous treatments, etc., were collected. Based on IL-6 levels. We divided the population into a high and low IL-6 group, comparing the efficacy and survival of the two groups.

All patients underwent a minimum of 2 cycles of PD-1 inhibitor therapy as per the drug’s guidelines, continuing until disease progression, intolerable toxicity, or at the patient’s request for termination. Treatment response was assessed using imaging techniques (CT or MRI) every two treatment cycles according to RECIST 1.1 criteria. Patients who were included underwent surveillance for disease remission and death and to record their progression-free survival (PFS) and overall survival (OS). PFS was defined as the amount of time from the start of PD-1 inhibitor therapy until the progression of the disease, death from any cause, or the follow-up cutoff date. OS was defined as the period of time between starting a PD-1 inhibitor treatment and either the follow-up cutoff date or death from any cause. The Huai’an Second People’s Hospital Institutional Review Board approved this study.

### Sample collection and measurement of serum cytokines

Blood samples were taken in EDTA tubes prior to patients receiving PD-1 inhibitor treatment. After being collected, all samples were processed within two hours and centrifuged for ten minutes at 4°C at 3000 rpm. The top plasma fraction was maintained at -80°C until analysis. The manufacturer’s instructions for measuring plasma IL-6 levels were followed when using an enzyme-linked immunosorbent assay (ELISA) kit with an anti-IL-6 antibody (nb600-1131, Novus).

### Tissue microarrays and immunohistochemistry

Tissue microarrays (TMAs) were procured from Shanghai Outdo Biotech Co., Ltd, and comprised tissues obtained from 110 esophageal cancer patients. The microarrays underwent incubation for 1 hour at 60°C (baked chips), followed by dewaxing in xylene twice and dehydration in graded alcohols, and then rinsed thrice with purified water. Subsequently, the chips were placed in a Citric acid antigen retrieval solution for antigen retrieval, followed by blocking with a blocking agent. The primary antibody used was rabbit polyclonal anti-IL-6 (Cat# nb600-1131, Novus), diluted in phosphate-buffered saline (PBS) at a ratio of 1:400. After diluting the microarray, the chip was first incubated with the main antibody for a whole night at 4°C. The chip was treated with an anti-rabbit secondary antibody for half an hour at room temperature following a PBS wash. Subsequently, diluted DAB was applied to the chip for color development. After rinsing, the slice was briefly stained with Haar Hematoxylin for 1 minute, dehydrated, air-dried at room temperature, and then sealed. Rabbit anti-human PD-L1 antibody (Cat# GT228002, GeneTech, China) and Rabbit anti-human CD8 antibody (Cat# PA577, Abcarta) were used to detect the expression levels of PD-L1 and CD8 in tissue microarrays, and the immunohistochemical steps were the same as above.

The PD-L1 Tumor Proportion Score (TPS) was used to measure PD-L1 expression. The formula used to determine the PD-L1 IHC score was: percentage of positive tumor cells × staining intensity ×100. Three groups based on PD-L1 expression levels were identified: <1%(low), 1-49% (moderate), and ≥50% (high). A three-tiered grading system based on cell density was used to assess the proportion of CD8+ lymphocytes to all nucleated cells in the stromal compartment: <1% (low), 1–10% (moderate), ≥10% (high). The proportion of positive tumor cells × staining intensity × 100 equals the IL-6 IHC score. The median score was used to categorize the results into two groups: high and low IL-6. Unaware of the patient’s clinical features, two pathologists independently evaluated each segment based on the evaluation criteria.

### Analysis of public databases

The TCGA database was used to get RNA-Seq data and clinical survival statistics for ESCC patients. TIMER 2.0 was used to analyze immune cell infiltration in the TCGA ESCC dataset (http://timer.cistrome.org) (accessed July 17, 2023), and the CIBERSORT algorithm is further verified. Using Spearman’s correlation coefficients, we assessed the level of correlation between immune cells infiltrating tumors and IL-6 gene expression.

### Statistical methods

Software such as GraphPad Prism 9 and SPSS 26.0 were used for statistical analysis. Frequencies and percentages of the count data were displayed. The Fisher’s exact or chi-square test was used to compare variables between groups. Kaplan-Meier survival curves were produced, and variations in survival were assessed using the log-rank test. The parameters that were independently associated with the patients’ OS and PFS were evaluated using Cox proportional hazards regression. Initially, a univariate analysis was performed, followed by including variables in the univariate analysis deemed clinically relevant and significant (P < 0.05) in a multivariable Cox regression model. Using R 4.2.1’s survminer package’s surv_cutpoint function, the optimal IL-6 cut-off value was ascertained. (https://www.r-project.org). Statistics were deemed significant when P <0.05.

## Results

### Relationship between IL-6 and tumor immunity

**Figure 1:**
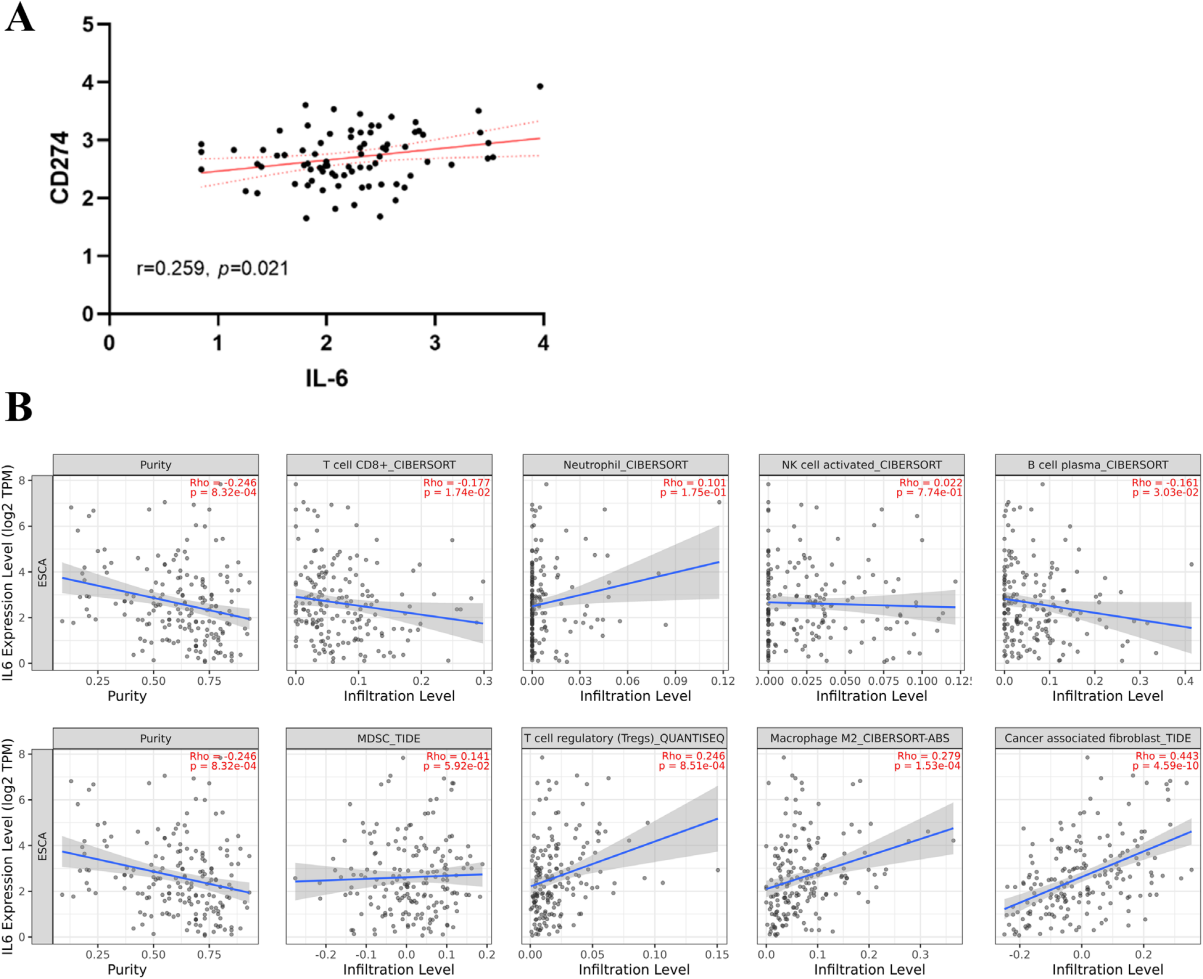
Relationship between IL-6 and tumor immunity. **(A)** Correlation between CD274 (PD-L1 transcription gene) and IL-6 expression in ESCC tumor tissues; **(B)** Correlation between tumor immune cells, immunosuppressive cells, and cancer-associated fibroblasts and IL-6 expression. The solid blue line indicates the regression curve, and the shaded area indicates the best-fit 95% confidence interval band.

**Figure 2:**
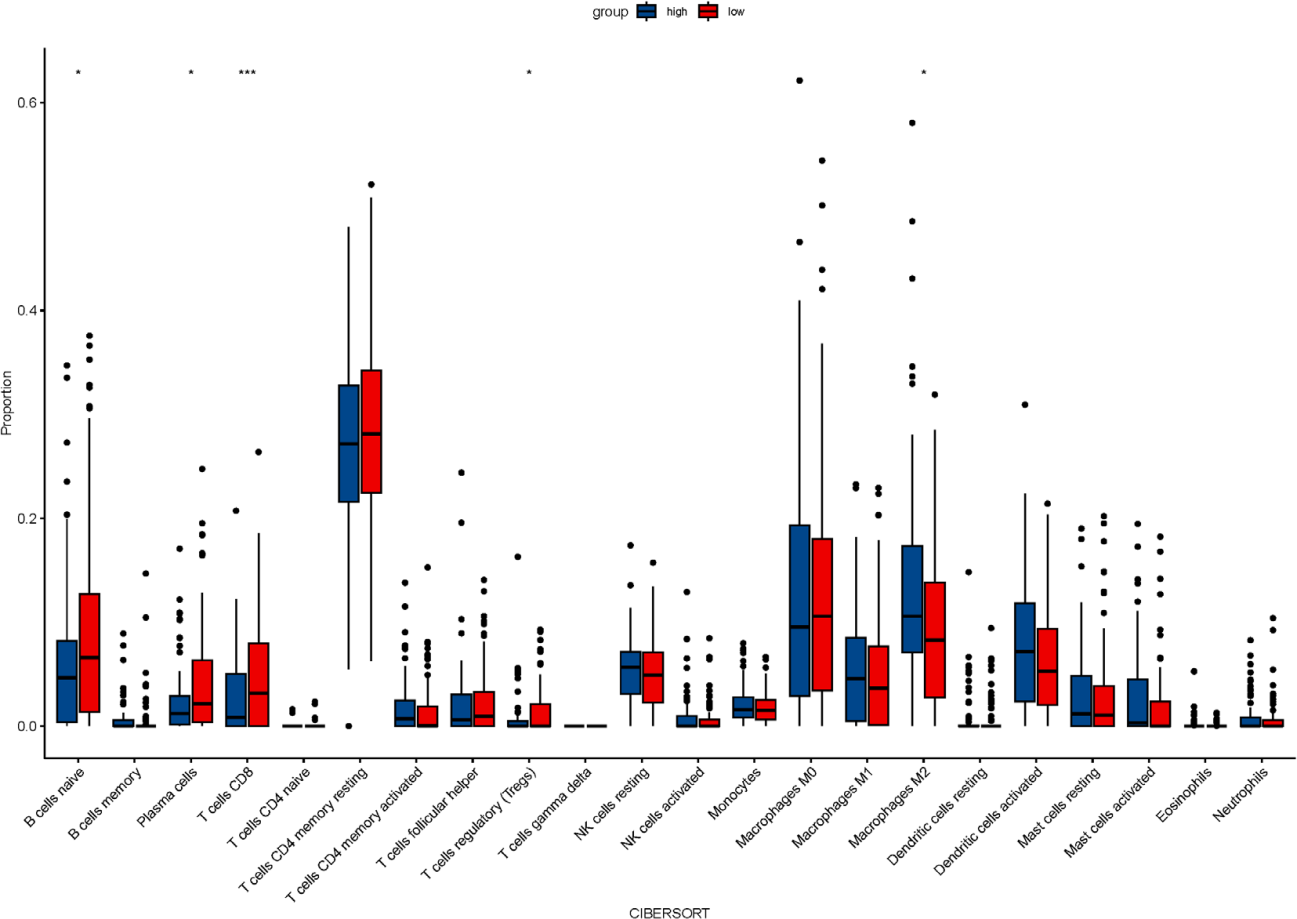
The relationship between IL-6 and 22 immune cells was calculated by the CIBERSORT algorithm.

From the TCGA database, transcriptome data for 79 ESCC patients was obtained. PD-L1, overexpressed on tumor cell surfaces, interacts with PD-1 on activated T cells, which causes cytotoxic T lymphocyte (CTL) dysfunction and T cell suppression, facilitating immune evasion and often correlates with poor clinical outcomes. A substantial positive correlation (r=0.259, P=0.021, **Figure 1A**) was found when we analyzed the connection between CD274 (PD-L1 transcription gene) and IL-6 mRNA levels. Using the TIMER 2.0 online database, we observed negative correlations of IL-6 expression with CD8+ T cells (r=-0.177, P=0.017) and B cells (r=-0.161, P=0.030) but positive correlations with immune-suppressing cells like M2 macrophages (r=0.279, P<0.001) and regulatory T cells (r=0.246, P=0.001). However, no significant correlations were found with neutrophils (r=0.101, P=0.175), NK cells (r=0.022, P=0.774), and MDSC cells (r=0.141, P=0.059) (**Figure 1B**). Consistent with previous findings, there was a notable positive association between IL-6 and cancer-associated fibroblasts (CAFs) (r=0.443, P<0.001, **Figure 1B**). Subsequently, we used the CIBERSORT algorithm to analyze more immune cells and obtained the same results; that is, IL-6 expression was negatively correlated with CD8 + T cells and B cells but positively correlated with immunosuppressive cells M2 macrophages and regulatory T cells. No significant correlation was found between IL-6 and other immune cells (**Figure 2**). Therefore, overexpression of IL-6 in the tumor tissues may contribute to immunosuppression within the tumor microenvironment of ESCC patients.

### Elevated IL-6 expression in ESCC tissues could be a predictive factor for ESCC patients

**Figure 3:**
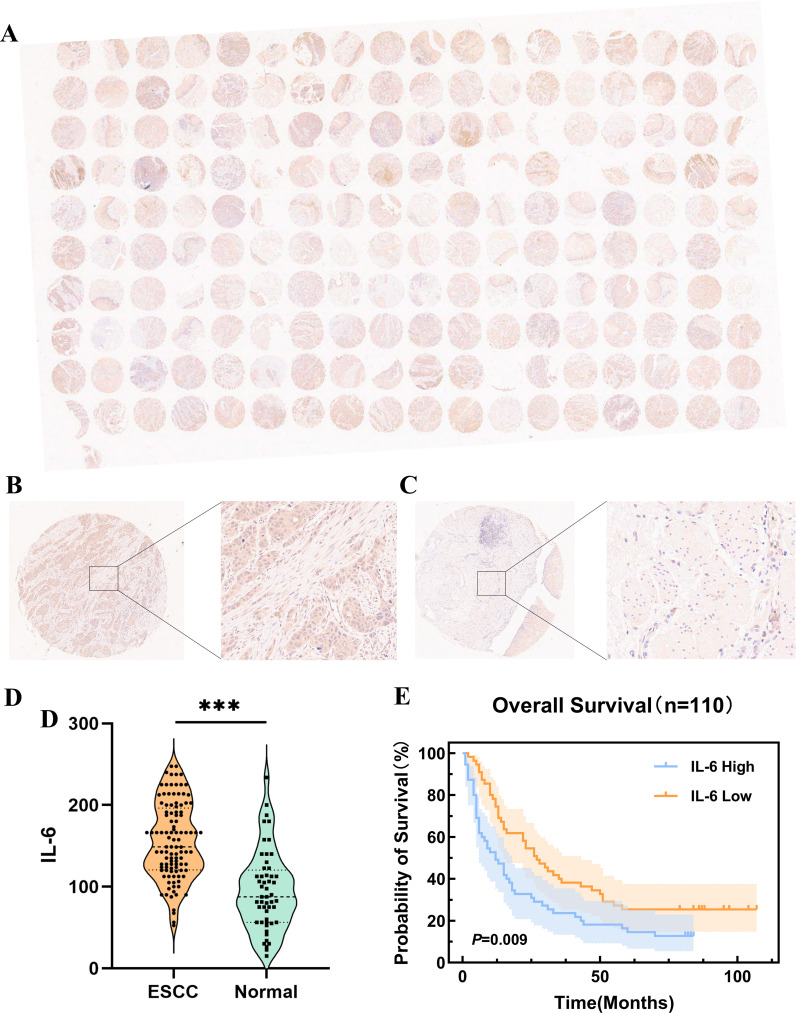
Expression of IL-6 in microarrays of ESCC tissues and correlation with prognosis. **(A)** Microarray scans of ESCC tissues after immunohistochemical staining for IL-6; IL-6 staining cancer tissues **(B)** and para-cancerous tissues **(C)** in ESCC TMAs; **(D)** Expression of IL-6 in ESCC tissues and para-cancerous tissues; **(E)** K-M survival curves of OS of ESCC patients based on IL-6 expression levels. ***P < 0.001.

IL-6 expression levels were assessed in ESCC TMAs (n=110) using immunohistochemistry (**Figure 3A**). The representative images of IL-6 staining tumor tissues (**Figure 3B**) and para-cancerous tissues (**Figure 3C**) in ESCC TMAs are shown in the figure. Compared to para-cancerous tissues, ESCC tissues exhibited significantly higher IL-6 expression levels (P < 0.001, **Figure 3D**), consistent with findings in other cancers. Patients were categorized into groups with low and high IL-6 levels depending on the median IL-6 expression in tumor tissues. When Kaplan-Meier survival curves were used to examine OS, it was shown that the group of high IL-6 had a shorter OS than the low IL-6 group (P=0.009, **Figure 3E**). The median OS values were 12.0 months (95% CI: 6.35-17.65) and 27.0 months (95% CI: 18.83-35.18). High levels of IL-6 in tumor tissues were found to be a risk element for ESCC patients’ prognosis by Cox regression analysis (HR=1.72, 95%CI: 1.13-2.61, P=0.011).

### Elevated IL-6 levels in ESCC tissues correlate with Type I (PD-L1+TILs+) tumors

**Figure 4:**
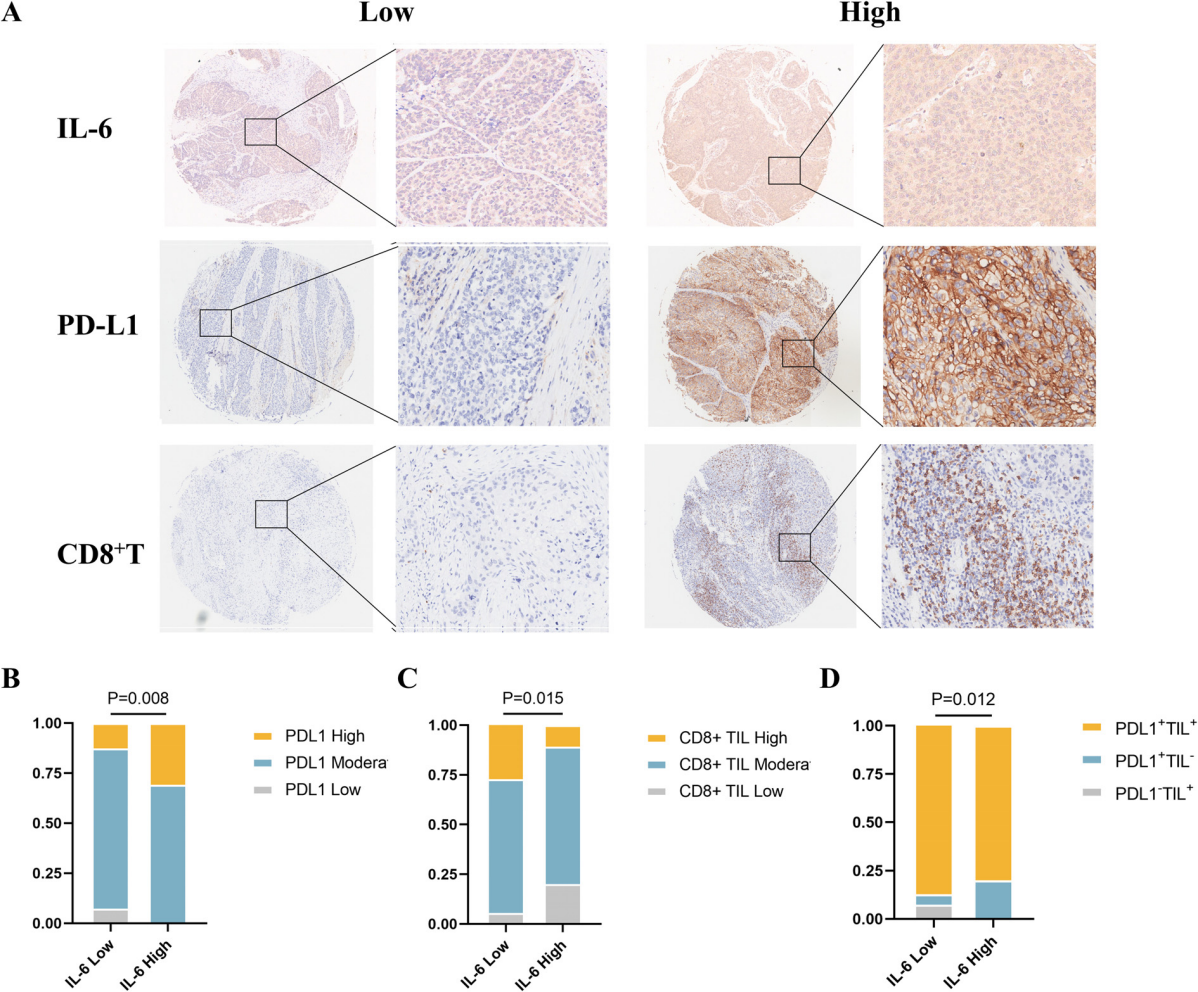
Relationship between IL-6 and tumor immune microenvironment type in ESCC tissues. **(A)** Representative samples stained for PD-L1, IL-6, and CD8 in ESCC TMAs; correlation of IL-6 expression levels in ESCC TMAs with PD-L1 expression **(B)**, CD8+ TIL infiltration **(C)**, and tumor immune microenvironment type **(D)**.

According to the expression of tumor-infiltrating lymphocytes (TILs) and PD-L1, Michele W. L. Teng et al. categorized the tumor microenvironment into four types, and the efficacy of ICI treatment can be initially assessed based on various tumor microenvironment types, allowing for a personalized treatment approach (17). Single-agent anti-PD-1/L1 blockade therapy is most likely beneficial for type I (PD-L1+ TILs+) cancers, which have been found to be highly responsive to immune checkpoint blockade therapy. Based on these results, we used immunohistochemistry to evaluate the expression levels of CD8+ T and PD-L1 cells in ESCC TMAs (**Figure 4A**). Our analysis revealed a higher incidence of high PD-L1 expression among patients with elevated IL-6 levels (P=0.008, **Figure 4B**), consistent with TCGA database results. Concurrently, CD8+ T cell infiltration was considerably lower in the high IL-6 group than in tissues in low IL-6 (P=0.015, **Figure 4C**), in line with the negative correlation observed in our TIMER 2.0 database. Additional analysis of the tumor microenvironment based on the expression of CD8+ T and PD-L1 showed that the high Compared to the low IL-6 group, the IL-6 group had a reduced percentage of type I (PD-L1+TILs+) tumors (P=0.012, **Figure 4D**). This suggests that elevated IL-6 expression may correlate with reduced responsiveness to immunotherapy in ESCC patients, potentially indicating a poorer prognosis.

### Patient characteristics and clinical outcomes

Using ELISA, we measured the pretreatment baseline plasma IL-6 levels in 141 patients with advanced ESCC receiving PD-1 inhibitor treatment at our institution. The cutoff value of 9.35 pg/ml for predicting PFS was determined using the surv_cutpoint function of the survminer package for R. Based on this cutoff value, the patients were categorized into high and low IL-6 groups, and **Table 1** shows their initial characteristics from two groups, which including age, gender, ECOG scores, smoking history, tumor stage, lesion site, metastatic organ involvement, history of radiotherapy and surgery, number of prior immunotherapy lines, type of PD-1 inhibitor used, and combination with chemotherapy and targeted therapy, showed no statistically significant differences between the groups (P > 0.05). Following RECIST1.1 evaluation criteria, patients underwent imaging assessments after every 2 treatment cycles to determine the best overall response (BOR): of the patients, 37 had progressive disease (PD), 79 had stable disease (SD), and 25 had a partial response (PR). The median PFS for all patients was 8.8 months (95% CI: 6.57-11.03), and the median OS was 22.6 months (95% CI: 11.11-34.03). **Table 1** displays patient characteristics.

**Table 1:** Baseline demographics of included patients with advanced ESCC.

Characteristic	Total (n=141)	IL-6 low (n=102)	IL-6 high (n=39)	χ 2	*P*
Age				0.778	0.378
<65	44 (31.2)	34 (33.3)	10 (25.6)		
≥65	97 (68.8)	68 (66.7)	29 (74.4)		
Gender				0.084	0.771
Male	95 (67.4)	68 (66.7)	27 (69.2)		
Female	46 (32.6)	34 (33.3)	12 (30.8)		
Smoking history				3.062	0.080
No	60 (42.6)	48 (47.1)	12 (30.8)		
Yes	81 (57.4)	54 (52.9)	27 (69.2)		
ECOG				3.012	0.083
0-1	85 (60.3)	66 (64.7)	19 (48.7)		
≥2	56 (39.7)	36 (35.3)	20 (51.3)		
Stage				0.031	0.860
III	49 (34.8)	35 (34.3)	14 (35.9)		
IV	92 (65.2)	67 (65.7)	25 (64.1)		
Tumor location				0.595	0.743
Cervical or upper thoracic	37 (26.2)	27 (26.5)	10 (25.6)		
Middle thoracic	62 (44.0)	43 (42.2)	19 (48.7)		
Lower thoracic	42 (29.8)	32 (31.4)	10 (25.6)		
Metastatic sites				0.692	0.405
<2	109 (77.3)	77 (75.5)	32 (82.1)		
≥2	32 (22.7)	25 (24.5)	7 (17.9)		
Previous radiotherapy				0.962	0.327
No	42 (29.8)	28 (27.5)	14 (35.9)		
Yes	99 (70.2)	74 (72.5)	25 (64.1)		
Previous surgery				0.377	0.539
No	92 (65.2)	65 (63.7)	27 (69.2)		
Yes	49 (34.8)	37 (36.3)	12 (30.8)		
Treatment lines				0.279	0.597
1	104 (73.8)	74 (72.5)	30 (76.9)		
≥2	37 (26.2)	28 (27.5)	9 (23.1)		
PD-1 inhibition agent				3.465	0.167
Sintilimab	34 (24.1)	27 (26.5)	7 (17.9)		
Camrelizumab	101 (71.6)	69 (67.6)	32 (82.1)		
Others(Toripalimab Pembrolizumab Penpulimab)	6 (4.3)	6 (5.9)	0 (0)		
Combined with other drugs				0.153	0.696
No	40 (28.4)	28 (27.5)	12 (30.8)		
Yes	101 (71.6)	74 (72.5)	27 (69.2)		
Best of response				6.703	0.035*
PR	25 (17.7)	21 (20.6)	4 (10.3)		
SD	79 (56.0)	60 (58.8)	19 (48.7)		
PD	37 (26.2)	21 (20.6)	16 (41.0)		

ECOG, Eastern Cooperative Oncology Group; PR, Partial Response; SD, Stable Disease; PD, Progressive Disease. *P < 0.05. Bold values represent meaningful p-values.

### Relationship between baseline plasma IL-6 and the prognosis of ESCC patients received with PD-1 inhibitors

**Figure 5:**
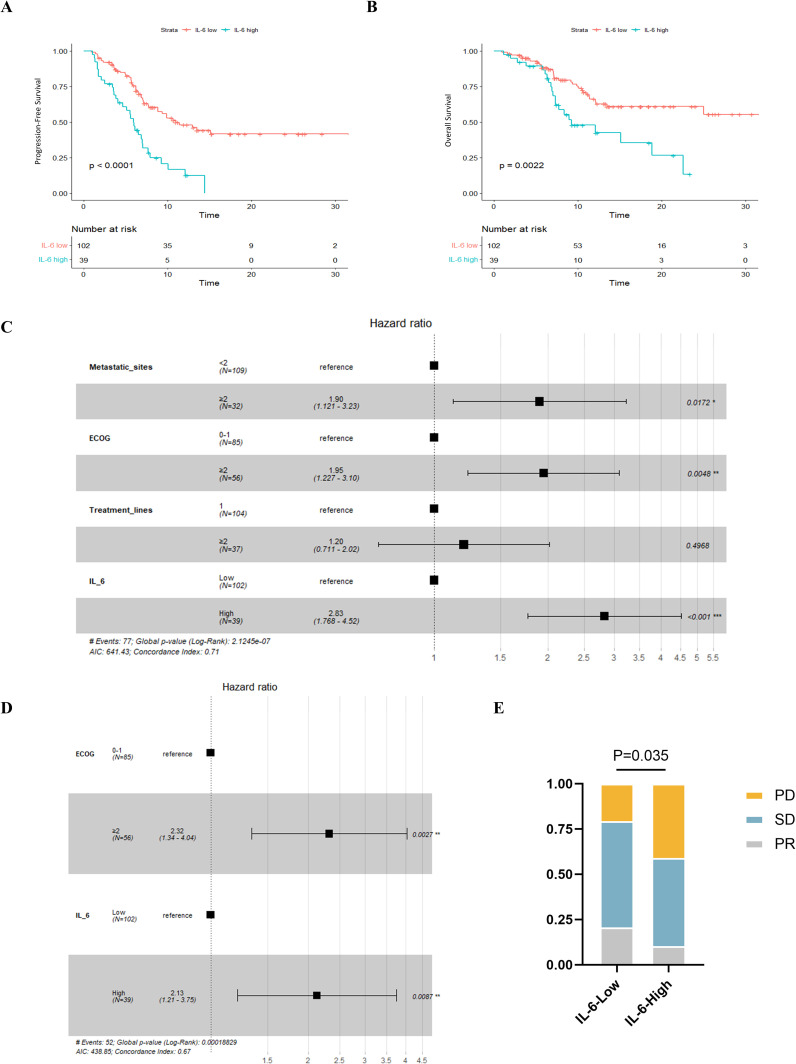
Relationship between plasma IL-6 levels and best of response and prognosis in patients with advanced ESCC. K-M survival curves for PFS **(A)** and OS **(B)** based on grouping of plasma IL-6 levels; forest plots showing the results of multifactorial analyses of PFS **(C)** and OS **(D)** performed by the number of metastatic organs, ECOG scores, the number of lines of immunotherapy, and plasma IL-6 levels; and **(E)** comparison of the best overall outcomes between the two groups of patients. ECOG, Eastern Cooperative Oncology Group; HR, Hazard Ratio; PR, Partial Response; SD, Stable Disease; PD, Progressive Disease.

When we examined the two groups’ survival results, we discovered that PFS (P < 0.001, **Figure 5A**) and OS (P = 0.002, **Figure 5B**) were poorer in individuals with greater IL-6 levels. The median PFS was 5.93 months (95% CI: 5.11-6.75) for patients with high IL-6 levels and 11.0 months (95% CI: 7.00-14.93) for those with low IL-6 levels. During our study period, individuals with high IL-6 levels had a median overall survival of 9.2 months (95% CI: 4.00-14.47), but individuals with low IL-6 levels did not attain a median OS.

Subsequently, we conducted univariate COX regression analysis of variables potentially influencing the prognosis of ESCC patients, including age, gender, number of metastatic organs, ECOG score, number of immunotherapy lines, chemotherapy or targeted therapy combination, and baseline plasma IL-6 levels. We discovered that the baseline plasma IL-6 levels, number of distant metastatic organs at the start of PD-1 inhibitor treatment, ECOG score, and number of treatment lines were associated with PFS in advanced ESCC patients. We calculated the correlation coefficients between each pair of variables using correlation analysis, all of which were less than 0.5, indicating no collinearity (**Table 2**). We then included these four variables in multifactorial COX regression analysis, revealing that high ECOG scores (HR=1.95; 95% CI: 1.23-3.10; P=0.005), more distant metastatic organs before treatment (HR=1.90; 95% CI: 1.12-3.23; P=0.017), and elevated plasma IL-6 levels (HR=2.83; 95% CI: 1.77-4.52; P<0.001) independently predicted poorer PFS in ESCC patients (**Table 3**, **Figure 5C**). Similarly, we identified associations between baseline IL-6 plasma levels and ECOG score with OS in patients with advanced ESCC through univariate analysis. Subsequently, these two variables were concurrently analyzed in a multivariate model, revealing that ECOG score (HR=2.32; 95% CI: 1.34-4.04; P=0.003) and IL-6 (HR=2.13; 95% CI: 1.21-3.75; P=0.009) independently predicted OS in ESCC patients (**Table 4**, **Figure 5D**).

**Table 2:** Coefficient of correlation for each pair of variables chosen using univariate analysis.

Correlation coefficient	Metastatic sites	ECOG	Treatment lines	IL-6
Metastatic sites	–	0.183	0.331	-0.07
ECOG	0.183	–	0.109	0.146
Treatment lines	0.331	0.109	–	-0.044
IL-6	-0.07	0.146	-0.044	–

ECOG, Eastern Cooperative Oncology Group.

**Table 3:** Univariate and multivariate analysis of factors influencing PFS in patients with advanced ESCC.

Variable	Category	Univariate		Multivariate	
		HR (95% CI)	*p*-value	HR (95% CI)	*p*-value
Age	<65 = 0;≥ 65 = 1	0.95 (0.60-1.52)	0.842		
Gender	Male=0; Female=1	0.86 (0.53-1.41)	0.559		
Metastatic sites	<2 = 0; ≥2 = 1	2.08 (1.29-3.36)	0.003*	1.90 (1.12-3.23)	0.017*
ECOG	0-1 = 0; ≥2 = 1	2.27 (1.45-3.56)	<0.001*	1.95 (1.23-3.10)	0.005*
Treatment lines	1 = 0; ≥2 = 1	1.60 (1.00-2.57)	0.049*	1.20 (0.71-2.02)	0.498
Combined with other drugs	No=0; Yes=1	1.03(0.62-1.71)	0.924		
IL-6	Low=0; High=1	2.80(1.76-4.47)	<0.001*	2.83 (1.77-4.52)	<0.001*

HR, Hazard Ratio; ECOG, Eastern Cooperative Oncology Group; *P < 0.05. Bold values represent meaningful p-values.

**Table 4:** Univariate and multivariate analysis of factors influencing OS in patients with advanced ESCC.

Variable	Category	Univariate		Multivariate	
		HR (95% CI)	*p*-value	HR (95% CI)	*p*-value
Age	<65 = 0;≥ 65 = 1	1.38 (0.76-2.49)	0.287		
Gender	Male=0; Female=1	0.80 (0.44-1.45)	0.456		
Metastatic sites	<2 = 0; ≥ 2 = 1	1.14 (0.62-2.09)	0.670		
ECOG	0-1 = 0; ≥2 = 1	2.51 (1.45-4.33)	0.001*	2.32 (1.34-4.04)	0.003*
Treatment lines	1 = 0; ≥2 = 1	1.52 (0.87-2.66)	0.143		
Combined with other drugs	No=0; Yes=1	0.94 (0.51-1.73)	0.836		
IL-6	Low=0; High=1	2.35 (1.34-4.12)	0.003*	2.13 (1.21-3.75)	0.009*

HR, Hazard Ratio; ECOG, Eastern Cooperative Oncology Group; *P < 0.05. Bold values represent meaningful p-values.

We also compared the BOR between the two groups of ESCC patients who received PD-1 inhibitors. Compared to the low IL-6 group, a greater percentage of patients in the high IL-6 group experienced disease progression (PD) (P=0.035, **Figure 5E**). The ORR (CR+PR) was 10.3% in the high IL-6 group, but it was 20.6% in the low IL-6 group, indicating that PD-1 inhibitor therapy had a better effect on patients whose baseline plasma IL-6 levels were lower.

## Discussion

The preliminary results from online databases and microarray exploration of esophageal cancer tissues suggest that IL-6 may regulate the tumor microenvironment and contribute to immunosuppression. High IL-6 expression appears to correlate with immunotherapeutic insensitivity. Our clinical study confirms that individuals with higher baseline plasma IL-6 experienced worse PFS, OS, and lower ORR. High IL-6 independently predicts unfavorable results in patients using PD-1 inhibitor treatment for advanced ESCC.

Currently, Less than 30% of ESCC patients receiving PD-1/PD-L1 blockers experience an overall remission rate, and although most patients initially respond to ICIs, acquired resistance can develop over time (18). Drug resistance is often related to an immunosuppressive tumor microenvironment (TME) and interactions between tumor cells and the TME. Therefore, it is crucial to identify reliable biomarkers that appropriately reflect the immune status of the tumor microenvironment.

The tumor immune microenvironment (TIME) comprises immune cells, tumor cells, and various cytokines, with their interactions dictating the trajectory of anti-tumor immunity. An immunosuppressive TME is a key barrier for tumor cells to achieve immune evasion, and immunosuppressive cells such as myeloid-derived suppressor cells (MDSCs), regulatory T cells (Tregs), and tumor-associated macrophages are abundant in it, and immunosuppressive molecules including PD-L1 and PD-1 are increased. Ming-Shao Tsai et al. (19) discovered a direct association between the IL-6 expression and PD-L1 in immunohistochemical studies of 248 tumor samples taken from individuals with squamous cell carcinoma of the head and neck. Further cellular tests confirmed that IL-6 affects the PD-L1 expression through the IL-6/JAK2/STAT3 pathway. The transcriptional data obtained from TCGA validated a direct association between IL-6 mRNA levels and PD-L1 expression in ESCC tumor tissues, and it was further validated by immunohistochemistry on ESCC TMAs. Specifically, a greater percentage of those with high IL-6 also had raised PD-L1 expression levels and subsequently experienced poorer OS, as observed in our ESCC tissue microarray immunohistochemistry results. Consequently, high IL-6 levels and up-regulated PD-L1 expression in ESCC tissues facilitated immune evasion by tumor cells.

Treg cells exhibit substantial immunosuppressive effects by regulating the activity of dendritic cells (DCs), NK cells, macrophages, and B cells through both humoral and intercellular processes (20). M2 macrophages promote tumor cell proliferation and metastasis by inhibiting T cell-mediated anti-tumor immune responses. The analysis conducted using the TIMER database demonstrated a direct association between IL-6 and M2 macrophages and Tregs in ESCC. Conversely, the expression of IL-6 exhibited an inverse association with the numbers of B cells and CD8+ T cells, also known as cytotoxic T lymphocytes, which have a vital function in the defense of malignancies by the immune system. Yosuke Ohno et al. (21) constructed an IL-6-deficient colon cancer mouse model and found significantly reduced tumor growth, which CD8+ T cell depletion eliminated. Under IL-6 deficiency, cytotoxic T cells accumulated at the tumor site, with more IFN-γ-producing T cells, indicating that IL-6 mediates CD8+ T cell exhaustion and affects their quantity. Furthermore, this study did not observe a statistically significant link between the NK cell numbers and IL-6. However, previous research has shown that IL-6 suppresses NK cell activity by downregulating surface-activated receptors such as NKp30 and NKG2D. reducing granzyme B secretion (22). As observed in prior studies, CAFs are significantly correlated with IL-6 levels in ESCC tissues. Takuya Kato et al. (23) demonstrated that CAFs influence intra-tumoral TIL (CD8+TIL and FoxP3+TIL) cells through IL-6 secretion. This indicates that the primary IL-6 producers in the ESCC tumor microenvironment are CAFs. IL-6 upregulates immunosuppressive cells, inhibits anti-tumor immune cells, or reduces their infiltration, constructing a highly immunosuppressive tumor microenvironment, potentially leading to ESCC progression and treatment resistance.

Tumor microenvironment types were first proposed by Michele W. L. Teng et al. Type I (PD-L1+TILs+) tumors exhibit pre-existing evidence of intra-tumor T cell inactivation due to PD-L1 binding, thus enhancing their PD-L1 blockade treatment responsiveness. We found a decreased proportion of Type I tumors in patients with high IL-6 compared to those with low IL-6 when the tumor microenvironment in ESCC TMAs was classified. This implies that in spite of the high level of PD-L1 expression, IL-6 may also contribute to the low response rate to immunotherapy in ESCC patients by mediating CD8+ T depletion, which prevents PD-L1 blockers from fully exerting their effects. Therefore, we initially hypothesized that IL-6 may be a prognostic indicator for ESCC patients undergoing ICIs.

Andressa S. Laino et al. showed that patients with malignant melanoma receiving ICIs had a poorer survival rate when they had greater levels of IL-6 at the beginning or increased IL-6 levels during treatment (24). Recognizing the role and potential of IL-6 in tumors, we collected blood specimens from 141 advanced ESCC patients before treating them with PD-1 inhibitors at our institution, and further analysis found that PFS and OS were lower in those with greater baseline IL-6 levels. and lower ORR. Subsequent COX regression analysis identified plasma IL-6 represents an independent risk factor for OS and PFS in advanced ESCC patients treated with PD-1 inhibitors after correcting for confounding variables, including the number of distant metastatic organs, ECOG scores, and treatment lines, which can be used to predict patient prognosis. Compared to currently used prognostic biomarkers, IL-6 can be directly detected in plasma without invasive sampling, making it easy to monitor dynamically and cost-effective. Therefore, it can be widely utilized in clinical practice to guide physicians’ decision-making.

ICI treatment resistance is often attributed to inadequate production and anti-tumor T cell function, compromised T cell memory development, and an immunosuppressive microenvironment with exhausted T cells, reducing the effectiveness of ICIs (25). Our study observed high IL-6 expression in ESCC, which correlated closely with immune cells and PD-L1 expression, contributing to a significantly suppressed tumor immune microenvironment. Blocking IL-6 and its downstream signaling pathway could potentially reverse this highly suppressed tumor immune microenvironment. Considering the close relationship between IL-6 and tumors, several drugs targeting IL-6/JAK/STAT3 signaling pathways are available on the market. These include IL-6 blocker stuximab, IL-6R receptor blocker tolizumab, JAK inhibitor ruxolitinib, and STAT3 inhibitors, which have been shown in preclinical experiments to significantly inhibit tumor growth, increase immune cell numbers, and improve the immunosuppressive microenvironment. Stuximab has demonstrated good clinical efficacy and safety in several clinical trials of prostate cancer (26, 27) and renal cancer (28, 29). The combined targeting of immune checkpoints and the IL-6/JAK/STAT3 signaling pathway represents a promising therapeutic strategy for ESCC patients with poor responses to ICIs. A preclinical investigation proved that inhibiting IL-6 effectively reestablished the mice’s resistance to anti-PD-L1 treatment in hepatocellular cancer models. Following combined IL-6 blockade, tumors exhibited improved responses to anti-PD-L1 therapy, leading to higher life periods and lower tumor sizes in mice (30). However, an open-label, multicenter, randomized Ib/II trial for platinum-refractory metastatic urothelial carcinoma did not demonstrate additional benefits in terms of ORR, PFS, or OS compared to atalizumab monotherapy, despite the tolerability of the combination treatment (31). This lack of benefit might be attributed to tumor heterogeneity and the involvement of IL-6 in multiple complex regulatory mechanisms, requiring validation in numerous clinical trials.

Compared with previous studies, the current research confirmed the association between baseline IL-6 and advanced ESCC patient prognosis and revealed IL-6’s close relationship with tumor immune cell expression and the immunosuppressive molecule PD-L1 within the tumor microenvironment. In ESCC, it was discovered that IL-6 regulated the immunosuppressive tumor microenvironment, partially explaining the poor reaction of immunotherapy in patients with elevated IL-6 levels. These findings also establish a foundation for a new therapeutic strategy that focuses on the IL-6 pathway and combines it with immunotherapy to potentially enhance treatment efficacy in ESCC.

There are various constraints to the investigation. Initially, the investigation was carried out as a retrospective study at a single center, potentially introducing bias that needs validation in a larger, multi-center cohort. Secondly, many patients receiving PD-1 inhibitors were concurrently treated with other medications, including second-line therapies, which could influence IL-6 levels. Although efforts were made to exclude patients during inflammatory periods, hidden inflammation or variations in liver function could still impact results. Lastly, while this study established an association between IL-6 and the ESCC tumor microenvironment, further basic experiments are necessary to elucidate IL-6’s specific regulatory mechanisms within the ESCC tumor immune microenvironment in the future.

In summary, our work affirms the strong connection between IL-6 and the immunosuppressive tumor microenvironment in ESCC. Our suggests that IL-6 may be a viable therapeutic target to improve the effectiveness of ICIs treatment in combating tumors. Furthermore, plasma IL-6 levels can serve as a biomarker to evaluate the effectiveness and prognosis of patients with advanced ESCC who are receiving PD-1 inhibitor therapy.

## Data Availability

The original contributions presented in the study are included in the article/supplementary material. Further inquiries can be directed to the corresponding author.
